# Factors associated with cancer pathogenesis among patients attending oncology clinic at Kamuzu Central Hospital in Malawi

**DOI:** 10.1186/1750-9378-7-S1-P9

**Published:** 2012-04-19

**Authors:** Agnes Moses, Elizabeth Bigger, Lindsey Wolf, Mike Owino, Albert Mwafongo, Maria Chikasema, Loreen Chiwoko, Mina Hosseinipour, Carol Shores

**Affiliations:** 1 Department of Medicine, UNC Project, Lilongwe, Malawi; 2 Department of Otolaryngology, University of North Carolina at Chapel Hill, Chapel Hill, NC, USA; 3 Department of Medicine, Division of Infectious Diseases, University of North Carolina at Chapel Hill, Chapel Hill, NC, USA; 4 Lineberger Comprehensive Cancer Center, University of North Carolina at Chapel Hill, Chapel Hill, NC, USA

## Introduction

Cancer is the cause of 13% of total worldwide mortality, and was the leading cause of in 2010 according to the World Health Organization. HIV prevalence in urban Malawi is approximately 20% and contributes to the pathogenesis of cancers, particularly AIDS-defining cancers. To evaluate risk factors for specific malignancies in Malawians, we designed an observational study to collect clinical data for cancer patients presenting at Kamuzu Central Hospital (KCH) in Lilongwe, Malawi.

## Methods

This was an observational study enrolled patients with suspected or confirmed malignancies presenting to Kamuzu Central Hospital (KCH) in Lilongwe, Malawi. From June 2010 to July, 2011, patients underwent interviews and medical chart reviews to complete database questionnaires. The questionnaire data were entered into a Web-based database and extracted into Microsoft Excel. Descriptive statistics were performed.

## Results

From June 2010 to July 2011 317 patients were enrolled into the study, 123 (39.3%) were male and 190 (60.7%) were female 4 had missing information. Age ranged from 18 to 86. 227 (70.9%) tested negative for HIV and 90 (28.3%) tested positive for HIV. 3 (0.94%) had missing HIV test results. 38 (11.9%) had Kaposi’s sarcoma, 12 (7.74%) had lymphoma, 71 (22.4%) had cervical cancer, 26 (8.2%) had breast cancer, 96 (30.2%) had esophageal cancer and the other cancers were smaller categories. 232 (76.32%) and 216 (71%) had never taken alcohol. Only 8.5% had family history of primary cancers. On past medical history, only 1.5% never had malaria, and 295 (93 %) reported to have past or present infection with malaria, TB, or schistosomiasis. Of note is that 89% of Kaposi’s sarcoma patients had concurrent HIV infection. Excluding the patients with KS, only 56 of the total were HIV positive.

## Conclusions

AIDS related malignancies are common in Malawi. However, HIV rates in traditionally non-AIDS related malignancies appear to match general population HIV prevalence rates.

